# Supplementary material for: Geographic and Ecological Diversity of Green Sulfur Bacteria in Hot Spring Mat Communities
Source: Microorganisms. 2023 Dec 4;11(12):2921. doi: 10.3390/microorganisms11122921 (PMC10746008; doi:10.3390/microorganisms11122921)
Supplement: Supplementary file 1 [file microorganisms-11-02921-s001.zip › microorganisms-2716425-supplementary.pdf]

Supplementary Information for

GEOGRAPHIC AND ECOLOGICAL DIVERSITY OF GREEN SULFUR BACTERIA IN HOT SPRING MAT COMMUNITIES

Donna L. Bedard[1], Greta Van Slyke[1], Ulrich Nübel[2,3], Mary M. Bateson[2], Susan Brumfield[4], Yong Jun An[1], Eric D. Becraft[2,5], Jason M. Wood[2,6], Vera Thiel[3], and David M. Ward[2] *

Sample collection and Processing

Media Preparation

Supplementary Table 1. Hot springs in which GSB were not found.

Supplementary Table 2. Diversity of bacteria found in the GSB Mat at site Lipayo-5.

Sample Collection and Processing

Yellowstone National Park:

Sampling in YNP was limited to summer for a variety of reasons including accessibility and weather. For this study we collected samples in YNP during the summers of 2000, 2001, and 2002 as described in Table 1 and Supplementary Table 1. Typically, we aseptically collected the samples and transferred them to microvials then onto ice for transport. Samples were stored at 4 °C and aliquots for later DNA analysis were stored at -20 °C, or long term at -80 °C. Samples were always tested by PCR with GSB-specific primers, (Table 2) within a few days of collection. Growth was only attempted with samples that tested positive with GSB-specific primers.

Big Spring samples from Gibbon Geysers Basin were collected twice in 2000, and once in 2001 on August 8. Sites BS1, BS2, and BS3 were sampled on June 21, 2000, and site BS3, an unusual floating mat, was sampled again on June 29; these samples were then designated BS3.1 and 3.2. The following year the mats at sites BS2 and BS3 were no longer there. Site BS1 was sampled again on August 6 and designated BS1-01.

MV samples from *Mud Volcano* were collected once each year from 2000 to 2002. As stated in the main text, these springs were ephemeral and did not persist from year to year. On June 29, 2000, small bubbling springs that were good candidates for GSB were discovered and sampled. We collected samples MV1 and MV2. It was already late in the day when we found them because we had already sampled at Big Spring and we were unable to explore further because it was getting dark. We visited this site again on August 7, 2001. We found and sampled MV3.

Sites MV1 and MV2 were no longer discrete springs. Now the site where they had been the previous year was a large mat spreading across the entire pool where they were located (Figure 3F); we collected samples MV4 and MV5. We visited the area for the last time on July 31, 2002, and collected sample MV6.

Mammoth Terraces was the easiest sampling site for us to visit and we visited it all three years. In all, we sampled the 8 sites listed in Supplementary Table 1 plus Narrow Gauge 2 and Angel Terrace listed in Table 1. Only the last two springs tested positive with GSB-specific primers, but enrichments from these samples did not grow. Angel Terrace was difficult to access and dangerous to sample, so we sampled there only once.

New Zealand: Mat samples were collected from Travel Lodge Spring in 1986 when DMW was there with other colleagues. When we discovered GSB in YNP hot springs in 2000 it seemed appropriate to revive these samples and to sequence the mat DNA which had never been sequenced before.

Lipayo 5, The Philippines: DLB had the opportunity to visit the Philippines in January of 2002 and took the opportunity to look for hot springs that might harbor GSB. Multiple sites were visited, but Lipayo 5 was the only site that showed evidence of possible GSB mats.

Sample processing: Samples were examined microscopically within a day or so after collection. Whenever samples tested positive by PCR we attempted to grow them as soon as possible. Only two water baths were readily available for these studies so our ability to test different temperatures was limited. Absorption spectra were done as soon as enriched cells were available. DNA extraction, cloning, and sequencing were done only after PCR results indicated the presence of GSB and as time allowed.

Media Preparation

The **Maintenance Medium** ([20] in main text) that we used for all cultures, including cultures revived from frozen stocks, was a modified version of Pfennig's Medium 29. In most cases we included thiosulfate which gave better growth. In enrichments derived from samples BS3.1 and BS3.2, thiosulfate encouraged the growth of purple sulfur bacteria that were also present in the mat, so we eliminated the thiosulfate for those enrichments.

For 250 ml combine in the order given:

Reduced, sterile ultrapure H_2O, 63.5 ml
1.5x Basal Salts Medium, 166.5ml
1M $NaHCO_3$, (42x) 5.95 ml
1.5 M MOPS (100x), 2.5 ml
100 mM Na_2S (40x), 6.25 ml
50 g/L $Na_2S_2O_3$ (50x), 5.0 ml (sodium thiosulfate, optional)
1000x SL10 (trace elements), 0.25 ml
5000x Vitamin B_{12} (100 µg/ml, stored frozen), 50 µl

To adjust pH we used a bromothymol blue indicator. On a small piece of parafilm, add one drop of the reagent and one drop of a buffer standard (from a set of standards made up at pH 6.7, 6.8, 6.9, and 7.0. Then add a drop of the medium to a drop of the reagent. Adjust medium by dropwise addition of sterile 2N NaOH or sterile 2N HCl until it matches the buffer standard at pH 6.9. When the medium reaches the correct pH, immediately dispense aseptically and under N_2 into sterile 17 ml screw top bottles filling each completely to the top.

1.5x Basal Salts Medium contains per liter deionized water:

EDTA, (free acid), 15 mg
$MgSO_4 \cdot 7 H_2O$, 0.3 g
$CaCl_2 \cdot 2 H_2O$, 75 mg
NaCl, 0.6 g
KH_2PO_4, 0.75 g
NH_4Cl, 0.6g
Ammonium acetate, 0.75 g (Omit to prepare photoautotrophic medium)

50X Sodium Thiosulfate:
Dissolve 10 g $Na_2S_2O_3 \cdot 5 H_2O$ in 200ml O_2-free ultrapure water and filter sterilize into an autoclaved bottle. Flush the bottle with sterile N_2 gas.

1 M Na HCO_3:
Dissolve 84.01 g in 1 L of boiled water that has been bubbled with CO_2. Gas the headspace with CO_2, seal tightly, and autoclave.

$Na_2S \cdot 9H_2O$:
Sodium sulfide gets oxidized in the bottle. Therefore, remove several crystals (about twice as much as needed) and wash with ultrapure water in a funnel lined with a paper filter. Dry the crystals in a hood and weigh out. Make up in O_2-free reduced ultrapure H_2O in a large serum bottle at 2.4018 g/100 ml. Seal with butyl rubber stopper and autoclave.
si
Trace elements Solution SL-10:
See https://www.dsmz.de/microorganisms/medium/pdf/DSMZ_Medium461.pdf

Bromothymol blue pH indicator:
Dissolve 20 mg of bromothymol blue in 3 ml of 0.01 NaOH. Add to 47 ml deionized distilled water.

Supplementary Table S1. Hot Springs where no Green Sulfur Bacteria were detected.

Mammoth Terraces Hot Spring Site	Date of Sample	Mat Description	Temp °C	pH	Sulfide Conc (μM)	GSB PCR product			GSB Enrichment
						600F	Nested 600F	619F	
Narrow Gauge 1	6/14/00<br>8/11/01<br>8/01/02	Dark olive green & orange fibrous mat growing upstream of white streamers	62.0<br>54.8<br>54.8	6.4<br>6.1<br>6.8	NT<br>30.4<br>14.9	No<br>No<br>NT	NT<br>No<br>NT	No<br>No<br>NT	NT<br>Negative
Painted Pool 1	6/14/00	Dark green fibrous mat	57.5	6.2	10.2[1]	No	NT	No	NT
Painted Pool 2	6/14/00	Green & orange slimy mat upstream of white streamers	58.2	6.3	24.1[1]	No	NT	No	NT
Cathy's Annex	6/14/00	Olive green& orange mat near white streamers	60.1	6.3	41.8[1]	No	NT	No	NT
Green Streamer	6/22/00	Green streamers	59.2	6.3	NT	No	No	No	Negative
New Mound	6/22/00<br>8/11/01<br>8/01/02	Olive green& orange mat upstream of white streamers	59.4<br>60.1<br>56.6	6.4<br>6.3<br>6.5	NT	No<br>No<br>NT	NT<br>No<br>NT	NT<br>No<br>NT	Negative<br>Negative
Painted Pool 3	6/22/00<br>8/11/01	Light pink & green fleshy mat on sides of vent above creamy white streamers	53.7<br>56.1	6.2<br>6.1	NT<br>25.4	No<br>No	No<br>No	NT<br>No	Negative
Painted Pool 4	6/22/00<br>8/11/01	Deep green fleshy mat collected under water on sides of a 25 cm deep vent	58.7<br>60.1	6.3<br>6.2	25.7[1]<br>7.9	No<br>No	No<br>No	NT<br>No	Negative

[1] Sulfide concentration for this sample was from a sample collected on 8/01/02 at a similar temperature and pH.

Supplementary Table S2. Diversity of eubacteria found in the green sulfur bacterial mat at site Lipayo 5.

# of Clones in this Classification	Clone Name	Sequence Length (bp)	Accession Number	RFLP Group	# of Clones in RFLP Group	Classification	% Identity	Phenotype	Further Classification
31	6A4<br>6B7<br>6G8<br>6N3<br>6F6<br>6B15	513<br>624<br>451<br>748<br>731<br>770	OQ913398<br>OQ913399<br>OQ913400<br>OQ913401<br>OQ913402<br>OQ913403	1<br>3<br>8<br>11<br>18<br>24	3<br>10<br>13<br>2<br>2<br>1	*Cyanobacteria*	100<br>100<br>100<br>100<br>100<br>100	Cyanobacteria<br>Oxygenic Photosynthesis	Could not be reliably classified beyond the Phylum Cyanobacteria because the classification is in flux.
17	6E3<br>6J3	1383<br>1382	OQ519263<br>OQ519264	12<br>5	9<br>8	*Chlorobaculum sp.*	98.5<br>98.6	Green sulfur bacteria<br>Anoxygenic phototroph	*Chlorobiales*<br>*Chlorobiaceae*
5	6G7	508	OQ913404	4	5	*Thiomonas* sp.	99.4	Sulfur/thiosulfate/sulfide oxidizer<br>Moderate acidophile	*Burkholderiales*<br>*Comamonadaceae*
3	6K3	813	OQ913405	17	3	*Hydrogenobaculum sp.*	100	Hydrogen-oxidizer<br>Thermoacidophilic<br>Thermolithotrophic<br>Requires elemental sulfur	Deeply branching<br>*Aquificales*<br>*Aquificaceae*
3	6K7<br>6B1	801<br>802	OQ913406<br>OQ913407	7<br>7	2<br>1	*Metallibacterium scheffleri*	99.6<br>99.8	Acidophile<br>Found in Acid mine drainage biofilm	*Lysobacterales*<br>*Lysobacteraceae*
3	6P10<br>6B11	685<br>729	OQ913408<br>OQ913409	14<br>22	2<br>1	*Geothrix* sp.	98.4<br>97.9	Fe III reducer<br>Acidobacteria.	*Holophagales*<br>*Holophagaceae*
1	6K5	737	OQ913410	48	1	*Desulfomonile* sp.	96.7	Sulfate/sulfur reducer	*Syntrophobacterales*<br>*Syntrophaceae*

Supplementary Table S2. Diversity of eubacteria found in the green sulfur bacterial mat at site Lipayo 5.

# of Clones in this Classification	Clone Name	Sequence Length (bp)	Accession Number	RFLP Group	# of Clones in RFLP Group	Classification	% Identity	Phenotype	Further Classification
1	6R4	747	OQ913411	58	1	*Massilia* sp.	98.0	Anaerobe<br>Root colonizing bacteria<br>Found in diverse environments	*Burkholderiales*<br>*Oxalobacteraceae*
3	6B2<br>6E8	728<br>733	OQ913412<br>OQ913413	2<br>32	2<br>1	Unclassified<br>*Campylobacterales*	95.0<br>97.0	Microaerophilic	*Epsilonproteobacteria*<br>*Campylobacterales*
1	6B12	756	OQ913414	23	1	Unclassified<br>*Ignavibacteriales*	92.4	Obligate anaerobe<br>Moderately thermophilic<br>Heterotroph	*Chlorobiota*<br>*Ignavibacteria*<br>Closely related to GSB
2	6A13	493	OQ913415	19	2	Unclassified<br>*Chitinophagaceae*	97.7	Facultative anaerobe	*Chitinophagia*<br>*Chitinophagales*
1	6J5	656	OQ913416	45	1	*Terrimicrobium* sp.	100	Anaerobe, nonmotile	*Terrimicrobiales*<br>*Terrimicrobiaceae*
